# Multidimensional poverty and catastrophic health spending in the mountainous regions of Myanmar, Nepal and India

**DOI:** 10.1186/s12939-016-0514-6

**Published:** 2017-01-18

**Authors:** Sanjay K. Mohanty, Nand Kishor Agrawal, Bidhubhusan Mahapatra, Dhrupad Choudhury, Sabarnee Tuladhar, E Valdemar Holmgren

**Affiliations:** 1 Department of Fertility Studies, International Institute for Population Sciences, Govandi Station Road, Deonar, Mumbai 400088 India; 2 International Centre for Integrated Mountain Development, Kathmandu, Nepal; 3 Population Council, New Delhi, India

**Keywords:** Multidimensional poverty, Catastrophic health spending, India, Nepal, Myanmar

## Abstract

**Background:**

Economic burden to households due to out-of-pocket expenditure (OOPE) is large in many Asian countries. Though studies suggest increasing household poverty due to high OOPE in developing countries, studies on association of multidimensional poverty and household health spending is limited. This paper tests the hypothesis that the multidimensionally poor are more likely to incur catastrophic health spending cutting across countries.

**Data and methods:**

Data from the Poverty and Vulnerability Assessment (PVA) Survey carried out by the International Center for Integrated Mountain Development (ICIMOD) has been used in the analyses. The PVA survey was a comprehensive household survey that covered the mountainous regions of India, Nepal and Myanmar. A total of 2647 households from India, 2310 households in Nepal and 4290 households in Myanmar covered under the PVA survey. Poverty is measured in a multidimensional framework by including the dimensions of education, income and energy, water and sanitation using the Alkire and Foster method. Health shock is measured using the frequency of illness, family sickness and death of any family member in a reference period of one year. Catastrophic health expenditure is defined as 40% above the household’s capacity to pay.

**Results:**

Results suggest that about three-fifths of the population in Myanmar, two-fifths of the population in Nepal and one-third of the population in India are multidimensionally poor. About 47% of the multidimensionally poor in India had incurred catastrophic health spending compared to 35% of the multidimensionally non-poor and the pattern was similar in both Nepal and Myanmar. The odds of incurring catastrophic health spending was 56% more among the multidimensionally poor than among the multidimensionally non-poor [95% CI: 1.35-1.76]. While health shocks to households are consistently significant predictors of catastrophic health spending cutting across country of residence, the educational attainment of the head of the household is not significant.

**Conclusion:**

The multidimensionally poor in the poorer regions are more likely to face health shocks and are less likely to afford professional health services. Increasing government spending on health and increasing households’ access to health insurance can reduce catastrophic health spending and multidimensional poverty.

## Background

In many developing countries, out-of-pocket expenditure (OOPE) is the primary means of financing health care. In the absence of a strong social security system, low-income level and insurance coverage, increasing longevity and non-communicable diseases in these countries, the OOPE on health care is large relative to household income. The high OOPE reduces the consumption of non-health goods and services of the households, disrupts the household level of living and pushes many families into the medical poverty trap and distress financing [1–3]. A large body of literature suggests that the OOPE on health care increases the extent of money-metric poverty [4–10]. Studies also suggest that the OOPE is catastrophic to poor, female headed households, households with an elderly member, rural households, large households, households with a chronically ill member and households without insurance [11–16].

The association of poverty and health is of interest across disciplines; among economists, sociologists, public health professionals, development practitioners and national and local governments. Studies drawing from these diverse fields have established a two-way causation of poverty and health; poverty is a major cause of ill health and ill health causes poverty [17–19]. Poverty is associated with lower longevity, higher infant and child mortality, higher maternal mortality, higher malnutrition, higher chronic diseases and higher burden of diseases among and within countries [20–23]. The association of income and health is strong at lower incomes [24]. Low health care utilization, poor social environment and environmental exposure, lack of availability of services and behavioral factors (the key proximate determinants of health) are positively associated with poverty [24–26].

A pro-poor health policy is high on the national and international development agenda because it plays a central role in human development and reducing poverty [27]. Investment in health increases household income, productivity, human capital, savings and economic growth and helps in demographic change [28, 29]. The global development agenda, the Millennium Development Goals (MDGs) and the Sustainable Development Goals (SDGs) highlight the inter-linkage of poverty and ill health and call for larger investment in health [30]. The synergy of poverty reduction and improvement in health has been priotorised by international organizations, bilateral donors, member nations and the local government [31].

Numerous studies have examined the role of socioeconomic status (SES) such as income, education and occupation in explaining inequality in health and health care utilization. The extent of health disparities varies by type of socioeconomic variable and is context specific [24, 32–35]. While the education gradient is a strong and consistent predictor of health, the economic gradient of health (measured by income or consumption or by wealth) is not consistent. Though many studies have examined the independent effect of SES on health and health care utilization, there are a few studies that examine the effect of multidimensional poverty on health. Using micro data from Japan, studies found that the composite measure of poverty is a good predictor of self-rated health, psychological stress and current smoking [36]. The multidimensionally poor are more likely to show health damaging behavior and chronic health conditions [37, 38]. Child survival is significantly lower among the multidimensionally poor than among the multidimensionally non-poor in India [39]. Though studies have examined the impact of money metric poverty and health shock, studies on multidimensional poverty and catastrophic health spending are limited. Given that poverty is a multidimensional construct and recent measurements focus on measuring poverty and vulnerability in a multidimensional space [40, 41], it is interesting to examine the association of multidimensional poverty, health, health care utilization and catastrophic health spending in the poorer regions of developing countries.

India, Nepal and Myanmar are three Asian countries that are unique with respect to poverty and catastrophic health spending. The percentage of the multidimensionally poor was estimated at 55% in India and 41% in Nepal [42] and the incidence of catastrophic health spending was high [1]. All three countries exhibit similar characteristics with respect to geography, economy and cultural practices, share borders and are at similar stages of demographic and epidemiological transition. The healthcare system in all the three countries caters to basic health services. Each country is characterized by a spatial disparity in the level of socioeconomic development and health care utilization. Drawing data from the poor regions in the mountainous areas of the three neighboring countries, India, Nepal and Myanmar, this paper demonstrates that the multidimensionally poor are more likely to have poor health, face health shock and incur higher catastrophic health spending compared to those who are multidimensionally non-poor. A systematic review and an analytical framework of poverty, ill health and health expenditure have been documented [43]. The paper has been conceptualized with the following rationale.

**First,** estimation of multidimensional poverty and catastrophic health spending is gaining attention. While people with multiple deprivations form a vulnerable segment of the population, catastrophic health spending reduces the extent of non-health consumption and indebtedness keeps them in the vicious circle of poverty. Hence, estimating multidimensional poverty and catastrophic health spending is a timely and useful exercise. **Second**, though a large body of literature links money-metric poverty with ill health, there is no study that examines the association of multidimensional poverty with health and health spending. People living with multiple deprivations are more likely to have poor health and incur catastrophic health spending because of their low income and lack of knowledge of the benefits of preventive and curative health care. **Third,** all the three countries are experiencing demographic and epidemiological transition. With reduction in fertility and increasing longevity, non-communicable diseases (NCDs) are the primary cause of morbidity and mortality. Treating NCDs are expensive and may increase the OOPE and catastrophic health spending. **Fourth**, this study provides a cross-country perspective of multidimensional poverty and catastrophic health spending in mountainous regions. The mountainous regions have lower accessibility to basic health services and are frequently affected by environmental hazards that may have direct bearing on the health of the population. These regions are also poor regions within their national boundary.

### Data

Data for the present study has been drawn from the Poverty and Vulnerability Assessment (PVA) survey carried out by the International Center for Integrated Mountain Development (ICIMOD) for India and Nepal during 2011–12 and for Myanmar during 2013–14. The aim of the PVA survey was to assess the livelihood vulnerability of the mountainous people in the Hindu Kush Himalayan (HKH)^1^ Region. These cross-sectional surveys are similar with respect to design, instrument and coverage in assessing the overall well being of mountainous people in each country. The PVA survey was carried out in seven districts in India (in the states of Assam and Arunachal Pradesh),^2^ six districts in Nepal (central and western regions) and eleven districts in Myanmar (Shan and Chin). All these districts selected are mountainous or Tarai (foothills) regions with poor accessibility to services. These districts were purposefully selected based on prior environmental hazards and representativeness in terms of ecological, ethnic, livelihood and socioeconomic aspects. Households were selected using a two-stage process. In the first stage, districts were stratified into several strata based on socioeconomic and ecological factors, and a pre-decided number of settlements were selected randomly from each strata. In the second stage, households were selected randomly from each selected settlement. The number of households per settlement surveyed was proportional to the total population. A total of 2310 households in Nepal, 2647 households in India were covered in 2011–12, while a total of 4290 households were covered in Myanmar during 2013 (Table 1). The household response rate was over 95% in each of the study areas. The study was not meant for national or regional estimates. The preliminary findings of these surveys are available in a report [44] and on the ICIMOD website. Using these data sets, some studies measured the vulnerability to climate, environmental and socioeconomic change in the HKH region [41] and multidimensional poverty for the selected districts of Nepal [45].

**Table 1:** Poverty and vulnerability assessment survey in India, Nepal and Myanmar - a sample profile of surveyed households and population

Variable	India	Nepal	Myanmar
Number of Households	2647	2310	4290
Average Household Size	5.69	5.71	5.42
Sex ratio (Male/female)	102	94	97
Percentage Urban	13.9	12.55	16.9
Percentage Literate	88.61	88.58	68.09
Monthly Per Capita Consumption Expenditure in US$	19.32	24.38	26.62
Percentage Population 0-14	33.61	33.30	30.42
Percentage Population 15-64	62.42	61.84	64.8
Percentage Population 65+	3.97	4.87	4.78

Assam and Arunachal Pradesh from where samples were drawn are two of the poorer states in India. About 41% of the population in Assam and 37% of the population in Arunachal Pradesh are living below the poverty line (higher than the national estimates of 28%) [46] and female literacy was estimated at 55% and 57% respectively compared to 65%, which is the national average. In the case of Nepal, the samples were drawn from the eastern and central regions of Nepal with a poverty head count ratio of 21% and 22% respectively, similar to the national average [47]. Female literacy in the eastern region was 59%, and 63% in the western region [47, 48] and much lower in the mountainous regions. In the case of Myanmar, eight districts from Shan and three districts from Chin were selected. About three-fourths of the population in Chin and 33% of the population in Shan lived below the poverty line in 2010 compared to the national average of 26% [49].

The PVA survey is a comprehensive survey that collected detailed information on economic well being (consumption expenditure, income, subjective economic well being) and social well being (education, health and health care), access to water, energy and sanitation and environmental shock of the surveyed households. Data on the consumption expenditure on food and non-food items were collected in a reference period of 30 days and 365 days. Health expenditure was collected in a reference period of 365 days. We have constructed a variable of monthly per capita consumption expenditure that does not include expenditure on tobacco, alcohol and food eaten outside. The non-food expenditure does not include health expenditure (as we are measuring catastrophic health spending), spending on social events and spending on agriculture. The total consumption expenditure is the sum of food and non-food expenditure. Regarding the health dimension, questions were asked to members about the frequency of illness during the twelve months preceding the survey. Similarly, a question was asked on the affordability of treatment for serious illness or injury to a household member on a six point scale (‘no’, ‘yes by borrowing’, ‘yes, with much difficulty’, ‘yes, with some difficulty’, ‘can afford’, ‘paid by employers’). The members were also asked to list the important shocks faced by the households during the twelve months preceding the survey, of which family sickness and death of a family member were two options. These variables are used in understanding the health shocks in the population.

## Methods

We have used the Alkire and Foster (AF) methodology to estimate the multidimensional poverty indices [40]. The three poverty indices, namely, the incidence of multidimensional poverty, the average intensity of poverty and the multidimensional poverty index are estimated for India, Nepal and Myanmar. Multidimensional poverty reflects the percentage of population who are deprived in the weighted deprivation score. The dimensions, variables and weight used in computing multidimensional poverty are given in Table 2.

**Table 2:** Dimensions, indicators and weight in the computation of multidimensional poverty index

Dimension	Indicators	National MPI indicators, Deprived	Weight	India	Nepal	Myanmar
				Mean deprivation		
Education	V1: Child schooling	Deprived if any school aged child (6–14) in the household is not attending school	0.17	0.04	0.04	0.24
	V2: Educational level of head of household	Deprived if the household head did not complete Class 5 or more	0.17	0.41	0.64	0.63
Living Standard	V3: Consumption poor	Deprived if the per capita income of the household is in the poorest quintile (bottom 20% of the distribution)	0.08	0.20	0.20	0.20
	V4: Asset Ownership	Deprived if the household does not own more than one radio, TV, mobile, telephone and does not own a motor cycle or car or truck	0.08	0.12	0.17	0.55
	V5:Housing	Deprived if both floor and roof are of grass/thatch/bamboo/plastic/tarpaulin/mud	0.08	0.15	0.12	0.13
	V6:Food Sufficiency	If the household does not have sufficient food for all 12 months	0.08	0.08	0.05	0.18
Energy, water and sanitation	V7: Electricity	Deprived if the household does not use electricity for lighting from Grid	0.11	0.49	0.19	0.83
	V8: Improved Drinking Water	Deprived if the household does not have access to improved drinking water or it takes 30 min or more to walk from home, round-trip for most of the year	0.11	0.37	0.50	0.24
	V9: Improved Sanitation	Deprived if the household uses open defecation or open pit	0.11	0.40	0.59	0.34

Three dimensions, namely, education, standard of living and access to basic services (water, sanitation and energy) are used in constructing multidimensional poverty. As our dependent variables are health shock and health expenditure, we have not included these variables in the estimation of multidimensional poverty. In the education domain, years of schooling of the head of the household and school attendance of children in the 6–14 age group are used. In the standard of living domain, we have used consumption expenditure, household assets, housing structure and food security. Consumption expenditure is a direct economic variable, which reflects the money metric poverty of households. We assigned equal weight to each dimension and equal weight to the variables within each dimension. There are three dimensions and a cut-off of 0.34 has been used to reflect multidimensional poverty; the estimates for different cut-off points (k) have been found to be robust.

Catastrophic health spending is measured using the methodology suggested by Xu [50]. A household is defined as incurring catastrophic health spending if the household’s health spending exceeds 40% of its capacity to pay. A brief description of estimating catastrophic health spending is given below.

1. Computation of poverty line (pl): The poverty line is defined as the average food consumption at the 45^th^ and 55^th^ percentiles of the total household expenditure of the respective countries.
2. Household subsistence expenditure (se_i_): poverty line*equivalent household size (eqsize_i_ = household size_i_^0.56^)
3. Capacity to pay (ctp_i_) = exp_i_-se_i_ if se_i_ < =foodexp_i_ and Capacity to pay (ctp) = exp_i_-food_i_ if se_i_ > food exp_i_

where exp_i_ is the expenditure of ith household, se_i_ is the subsistence expenditure of ith household, food_i_ is the food expenditure of ith household.

A household is said to incur catastrophic health expenditure if

OOPE_i_/ctp_i_ > =0.4

where OOPE_i_ is the out-of pocket expenditure on health and

ctp is the capacity to pay of i^th^ household.

Four health related variables included in the analyses are

1. family sickness and death of family members during the year preceding the survey
2. health shock as a major shock in the household
3. affordability of professional health care, and
4. catastrophic health spending.

## Results

**Fig. 1:**
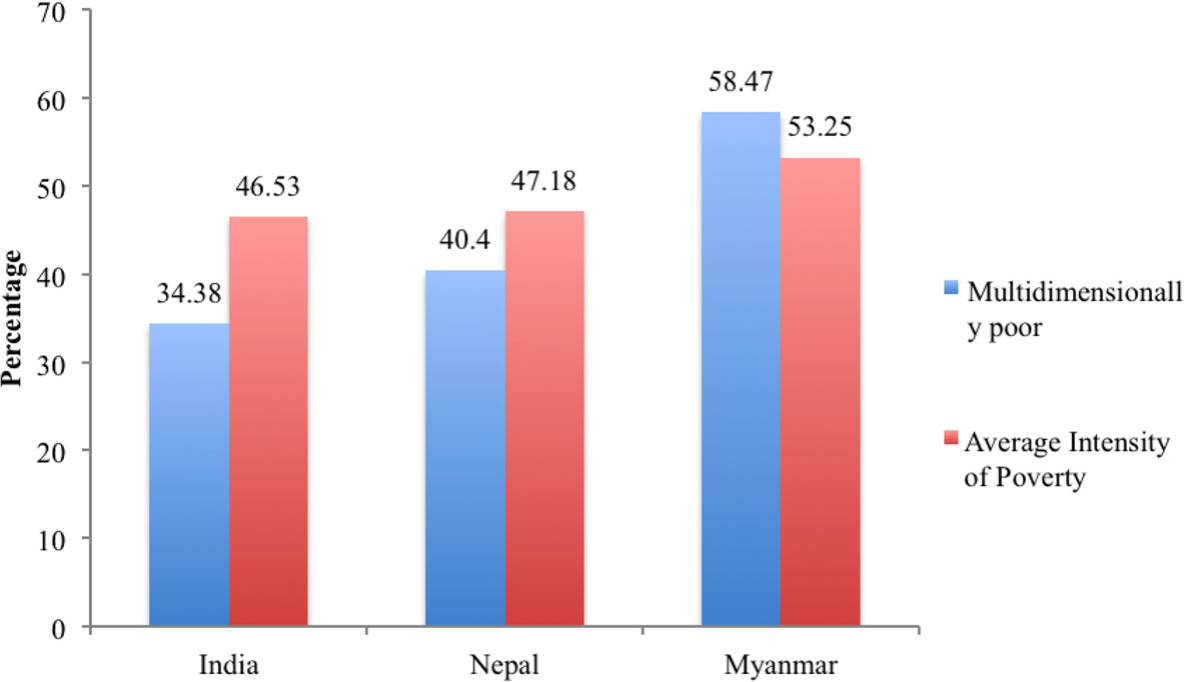
Percentage of Multidimensionally Poor and the Average Intensity of Poverty in a Sample Population of India, Nepal and Myanmar, 2011–13

Figure 1 presents the extent of multidimensional poverty and the average intensity of poverty in the study population of India, Nepal and Myanmar. In our sample, about two-thirds of the population were multidimensionally poor in Myanmar, two-fifths in Nepal and one-third in India. The average intensity of poverty (on average, the multidimensionally poor are deprived) was 53% in Myanmar and 47% each in India and Nepal. The multidimensional poverty index (MPI) was 0.31 in Myanmar, 0.22 in India and 0.16 in Nepal suggesting the varying deprivations in these regions.

### Multidimensional poverty and health shock

Table 3 presents the differentials in monthly per capita consumption expenditure (MPCE), monthly per capita health spending and health expenditure as percentage of per capita consumption expenditure along with a number of health variables among the multidimensionally poor and non-poor. The MPCE reflects the economic well being of the household. As expected, the MPCE was higher among the multidimensionally non-poor than among the multidimensionally poor in all the countries. The per capita health spending was lower among the multidimensionally poor than among the multidimensionally non-poor but per capita health spending as a percentage of MPCE was higher among the multidimensionally poor than among the multidimensionally non-poor in each of these countries. For example, in India, per capita health spending accounts for 14.8% of MPCE among the multidimensionally poor compared to 13.9% among the multidimensionally non-poor.

**Table 3:** Monthly Per Capita Consumption Expenditure, Health Expenditure as Percentage of Consumption Expenditure, Frequency of Illness of Household Members and Affordability of Health Services among the Multidimensionally Poor and the Multidimensionally Non-Poor Households in a Sample Population of India, Nepal and Myanmar, 2011-13

Variable	India			Nepal			Myanmar		
	Multidimensionally poor	Multidimensionally non-poor	All	Multidimensionally poor	Multidimensionally non-poor	All	Multidimensionally poor	Multidimensionally non-poor	All
Monthly per capita Consumption Expenditure (MPCE) in US$	14.37	21.92	19.32	21.18	26.55	24.38	22.27	32.76	26.62
Monthly per capita health expenditure in US$	2.02	3.32	2.88	3.14	3.69	3.47	2.35	3.42	2.79
Monthly per capita health expenditure as percentage of MPCE	14.84	13.91	14.23	10.55	10.44	10.49	10.55	10.44	10.49
Frequency of illness of any household member during the 12 months preceding the survey									
Never/once/twice a year	56.08	66.93	63.20	75.42	78.03	76.98	91.80	92.38	92.03
Once or more than once in a month	43.92	33.07	36.80	24.58	21.97	23.02	8.20	7.63	7.96
Affordability of professional treatment									
Cannot afford	61.75	46.64	51.83	59.79	44.36	50.60	17.66	7.93	13.62
Can afford with difficulty	31.46	35.32	33.99	20.76	17.35	18.73	41.14	28.07	35.72
Can afford	6.79	18.05	14.18	19.45	38.28	30.68	41.20	64.00	50.67
Shocks/Problems faced by household members during the 12 months preceding the survey									
Family Sickness	68.12	57.89	61.41	49.78	41.14	44.63	50.25	52.47	51.17
Death of Household Members	4.97	4.24	4.62	5.01	4.36	4.49	2.65	2.56	2.64
Number of Households	854	1793	2647	960	1350	2310	2514	1776	4290

On the other hand, the health shocks were more among the multidimensionally poor than among the multidimensionally non-poor, irrespective of the country of residence. In India, in about 44% of the multidimensionally poor households, a household member faced illness once in a month or more than once compared to 33% of the multidimensionally non-poor. On the other hand, majority of the households among the multidimensionally poor could not afford to meet their health expenses and had to borrow compared to the multidimensionally non-poor in all the three countries. Similarly, a higher proportion of the households among the multidimensionally poor reported family sickness as one of the major health shocks during the twelve months preceding the survey compared to the multidimensionally non-poor in India and Nepal, while the differences were small in Myanmar. The incidence of death of any family member among the multidimensionally poor was significantly higher than that among the multidimensionally non-poor, irrespective of place of residence (4.97% vs. 4.27% in India; 5.01% vs. 4.36% in Nepal and 2.65% vs. 2.56% in Myanmar). These results confirm that the multidimensionally poor face higher health shocks and incur higher OOPE than their ability to pay in these countries.

### Multidimensional poverty and catastrophic health spending

**Fig. 2:**
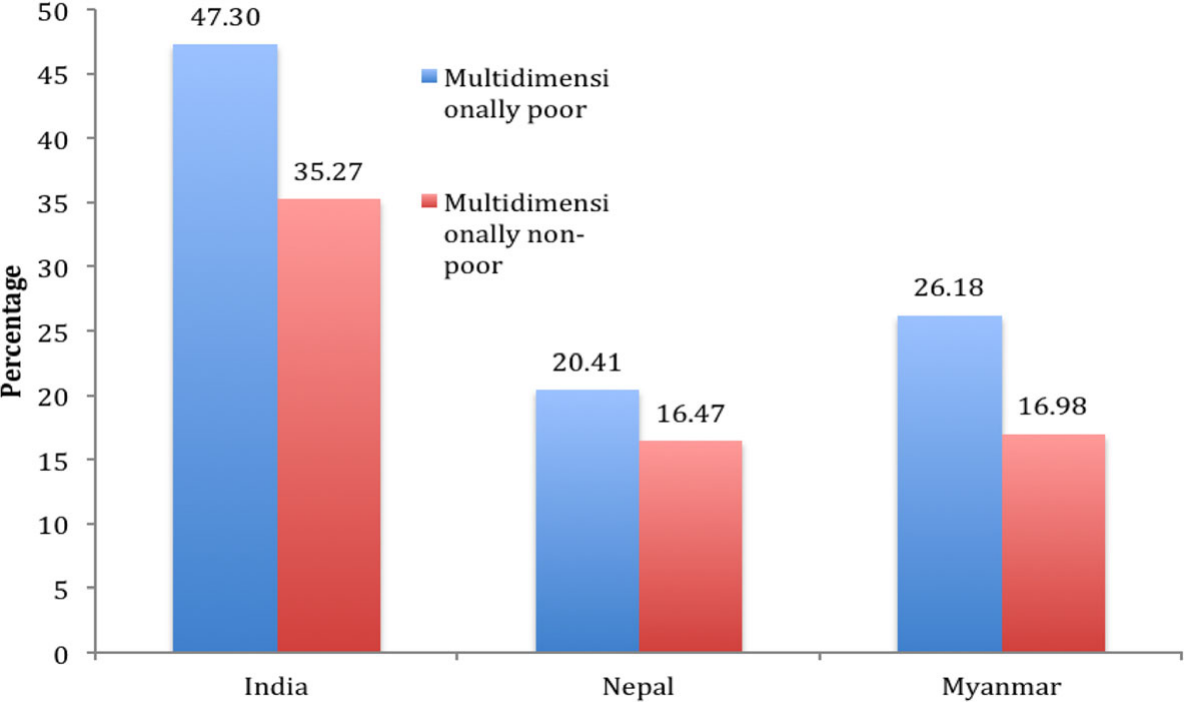
Percentage of Population Incurring Catastrophic Health Spending by Multidimensional Poverty in a Sample Population of India, Nepal and Myanmar, 2011–13

Here, we examine the differentials in catastrophic health spending among the multidimensionally poor and multidimensionally non-poor households controlling for selected demographic characteristics of the head of households and household economic variables. Figure 2 presents the extent of catastrophic health spending among the multidimensionally poor and non-poor in the three countries. The incidence of catastrophic health spending was significantly higher among the multidimensionally poor compared to the multidimensionally non-poor, irrespective of the country of residence. In India, 47% of the multidimensionally poor households incurred catastrophic health spending compared to 35% of the multidimensionally non-poor. The pattern of catastrophic health spending was similar in Nepal and Myanmar though the extent varied. Among the three countries, the incidence of catastrophic health spending was highest among the multidimensionally poor in India and least among the multidimensionally non-poor in Nepal with 20.41% and 16.47% respectively. In Myanmar, catastrophic health spending among the multidimensionally poor was 26% compared to 17% among the multidimensionally non-poor.

Table 4 presents the differentials in catastrophic health spending by characteristics of the head of household. With respect to age, there is no pattern in catastrophic health spending by multidimensional poverty status within and across countries. The extent of catastrophic health spending was also higher among the female headed households and multidimensionally poor than among the female headed households who were multidimensionally non-poor in all the three countries. The incidence of catastrophic health spending was higher among the multidimensionally poor than among the multidimensionally non-poor for both currently married and ever married/never married in all the three countries. In India, the extent of catastrophic health spending among the illiterate belonging to multidimensionally poor households was the highest (52%) and the least among those who had eleven years of education and more. Catastrophic health spending declines with consumption quintile irrespective of their place of residence suggesting that the economically better off households are less likely to incur catastrophic health spending. The extent of catastrophic health spending among the multidimensionally non-poor was lower than that among the multidimensionally poor in all the three countries. The incidence of catastrophic health spending was higher in rural areas than in urban areas.

**Table 4:** Percentage of Population Incurring Catastrophic Health Spending by Multidimensional Poverty and Household Characteristics of Sample Households in India, Nepal and Myanmar, 2011-13

Variable	India			Nepal			Myanmar		
	Multidimensionally poor	Multidimensionally non-poor	All	Multidimensionally poor	Multidimensionally non-poor	All	Multidimensionally poor	Multidimensionally non-poor	All
Age of Household Head									
Lt31	35.29	47.42	42.77	21.02	24.98	23.49	31.09	19.44	26.21
31-45	46.40	30.97	36.61	15.04	14.16	14.48	25.83	16.69	22.10
46-60	46.85	34.16	38.40	25.01	14.33	18.77	24.70	16.46	21.24
61+	56.70	40.57	45.51	19.44	24.05	21.88	23.95	15.52	20.44
Sex of the Head of the Household									
Male	46.97	35.00	39.04	21.51	16.35	17.89	23.99	13.93	20.08
Female	49.98	38.17	42.96	20.23	19.48	20.43	29.77	20.77	25.67
Marital Status of the Head of the Household									
Currently married	46.36	34.97	38.79	20.10	18.94	17.92	24.80	16.72	21.56
Otherwise	54.82	38.93	45.72	22.91	16.52	21.06	35.71	18.18	26.96
Educational level of the Head of the Household									
Illiterate	51.62	38.57	47.39	21.61	18.02	20.41	22.49	12.44	20.37
Literate formal/less than class 5	43.95	41.26	42.98	17.96	14.39	16.21	29.28	10.82	24.10
Class 5-7	46.93	40.86	41.86	26.35	23.16	23.36	34.35	24.46	27.87
Class 8-10	44.75	35.38	36.48	23.96	11.35	11.96	45.84	19.16	25.49
11+ years/professional	17.40	26.56	26.00	11.27	16.76	16.60	15.17	10.90	11.45
Consumption Quintile									
Poorest	52.42	58.49	54.10	34.46	37.80	35.37	45.18	40.14	44.47
Poorer	52.21	44.82	47.44	18.43	21.07	20.00	29.31	31.61	30.08
Middle	47.71	41.65	43.26	22.93	15.93	18.58	15.67	15.06	15.43
Richer	40.61	34.44	36.02	10.25	16.95	14.69	13.52	12.57	12.98
Richest	36.51	18.75	22.78	10.78	9.93	10.19	7.91	9.27	8.78
Place of Residence									
Rural	59.45	23.89	25.74	21.37	18.65	19.85	23.22	16.77	18.17
Urban	47.80	37.63	41.24	6.84	8.41	8.11	26.37	17.07	23.17
Number of Households	854	1793	2647	960	1350	2310	2514	1776	4290

### Determinants of catastrophic health spending

Table 5 presents the odds ratio and significance level of catastrophic health spending for all three countries (combined) and for each country separately. We have included multidimensional poverty, the country of residence, sickness and death of family members along with household characteristics as predictors in the model. Model 1 presents the pooled result of three countries, model 2 presents for India, model 3 for Nepal and model 4 for Myanmar. The multidimensionally poor are 54% more likely to incur catastrophic health spending compared to the multidimensionally non-poor in the pooled model. In the case of a country specific model, multidimensional poverty is significant in India and Myanmar, but not in Nepal. The educational level of the head of household does not show any consistent result across groups and countries. Rural households are more likely to face catastrophic health spending compared to urban households, irrespective of country of residence. The variables on health shock are significant across countries and in the combined model. Households that reported illnesses of a member at least once a month are significantly more likely to incur catastrophic health spending compared to those households where members do not fall sick frequently. Similarly, households that experienced the death of a family member in the twelve months preceding the survey were more likely to incur catastrophic health expenditure. This is also true of cases where sickness of a member was reported as a major shock, irrespective of the country of residence. With reference to the three countries, the odds of incurring catastrophic health spending among households in India is 2.3 times more than that of Nepal, and that of Myanmar is 1.6 times more than that of Nepal. This finding is comparable to the data on the overall poverty level in India.

**Table 5:** Determinants of Catastrophic Health Spending in India, Nepal and Myanmar, 2011-13

	Model 1 (Combined)		Model 2: India		Model 3: Nepal		Model 4: Myanmar	
	Odds Ratio	95% CI	Odds Ratio	95% CI	Odds Ratio	95% CI	Odds Ratio	95% CI
Multidimensionally non-poor (R)								
Multidimensionally poor	1.54***	1.35-1.76	1.76***	1.17-2.04	0.93	0.70-1.23	1.80***	1.50-2.18
Age (<31- R)								
31-45	0.76***	0.64-0.90	0.9	0.51	0.53***	0.35-0.81	0.81	0.65-1.01
46-60	0.85*	0.72-1.01	1.01	0.5	0.65*	0.43-1.00	0.97	0.77-1.21
61-98	1.09	0.89	1.33	0.74	0.84	0.53-1.35	1.19	0.90-1.57
Sex of the Head of the Household (male- R)								
Female	1.09	0.95	1.24	0.81	1.11	0.78-1.60	1.07	0.92-1.26
Marital Status of the Head of the Household (Currently married- R)								
Others	1.21**	1.03-1.43	1.43	0.56	0.96	0.63-1.45	1.36***	1.11-1.65
Educational level of the Head of the Household (11–15 Years of schooling reference)								
Illiterate	0.83**	0.69-0.99	0.99	0.75	1.57*	0.99-2.0	0.60***	0.47-0.77
Less than 5	0.75***	0.62-0.91	0.91	0.54	1.1	0.69-1.74	0.63***	0.47-0.83
5-7	1	0.84	1.21	0.65	1.42	0.87-2.32	0.84	0.65-1.09
8-10	0.77	0.63	0.94	0.6	1.43	0.87-2.33	0.56***	0.39-0.79
Residence (Urban-Reference)								
Rural	1.40***	1.19-1.63	1.63***	1.24-2.42	1.62	1.11-2.35	1.26**	1.03-1.55
Illness of a member of the household at least once a month (None-Reference)								
At least a member of the household was sick	2.12***	1.88-2.39	2.39***	1.95-2.98	1.09	0.84-1.41	2.51***	2.09-3.00
Death of a member in the family in the year preceding the survey (None-Reference)								
Household experience death of one or more member	7.65***	5.85-10.01	10.01***	5.02-13.82	5.94***	3.54-9.97	8.81***	5.81-13.37
Sickness of a member reported as major shock (None-Reference)								
Household experienced health shock	7.44***	6.53-8.47	8.47***	10.62-18.91	7.32***	5.49-9.75	5.64***	4.73-6.72
Country (Nepal-R)								
India	2.20***	1.89-2.57	2.57					
Myanmar	1.65***	1.42-1.92	1.92					
Constant	0.04***	0.03-0.05	0.05***	0.02-0.07	0.04***	0.02-0.08	0.08***	0.06-0.11

**** *p* <0.001 ** *p* < .01 * *p* < 0.1

## Discussion

Education and health are two important aspects of human capital that are accorded high priority in the national and international development agenda. Though many Asian countries continue to make investments in education and health, the healthcare system in these countries mainly offers basic health services. For example, in India, two-thirds of national health spending is on maternal and child health. A pro-poor health system is now a prerequisite of many national governments. India, Nepal and Myanmar, are three Asian countries with typical cases of experiencing increasing longevity, increasing non-communicable diseases (NCDs), high out-of-pocket expenditure on health care and low ability to meet health care needs. Majority of the population in these countries meet the health care needs through out-of-pocket spending. A large number of studies established high poverty due to OOPE in these countries [1, 5, 9]. While the poor are certainly disadvantaged in seeking health care, many non-poor households are reducing non-food expenditure, have reduced access to health care and are prone to long-term impoverishment. It is particularly disadvantageous to those who face multiple deprivations, have low educational attainment, low ability to pay and poor access to water, sanitation and energy. Multidimensional poverty captures the multiple deprivations and is increasingly used in poverty analyses and policy. In this context, this paper tests the hypothesis that the multidimensionally poor are more likely to face health shocks and catastrophic health spending in the poorer regions of developing countries. We have used the PVA survey data that used similar instruments and the best practice methodology to measure multidimensional poverty and catastrophic health spending. Our findings illustrate multidimensional poverty and catastrophic health spending in the poorer and mountainous regions in Asia and provide a comparison between the multidimensionally poor and non-poor. However, we make no generalizations on multidimensional poverty and catastrophic health spending regarding any country.

### We have some interesting findings

First, the extent of multidimensional poverty in the study population of India, Nepal and Myanmar was 34%, 40% and 58% respectively. The global estimate of multidimensional poverty was 55% in India (2005–06), 41% in Nepal (2011) [42] and not available for Myanmar. The national estimates of multidimensional poverty in India are a decade old and not comparable with our estimates and the estimates of Nepal are close to our estimates. The consumption poverty was higher in India than in Nepal, while the multidimensional poverty was higher in Nepal than in India. This is perhaps because the non-economic dimensions such as education and access to basic facilities are relatively poorer in Nepal.

Second, the multidimensionally poor were less likely to afford professional health services compared to the multidimensionally non-poor. This can be linked to health shocks such as higher number of deaths of family members among the multidimensionally poor than among the multidimensionally non-poor, irrespective of the country of residence. The extent of illness was also higher among the multidimensionally poor in India and Nepal. It confirms that the poor bear the health burden disproportionately. Moreover, these regions often experienced environmental shocks, which may be linked to the poor health of the population.

Third, the OOPE on health was significantly higher among the multidimensionally poor than among the multidimensionally non-poor.

Fourth, the incidence of catastrophic health spending was higher among the multidimensionally poor than among the multidimensionally non-poor, irrespective of the country of residence. This may be due to a multiplicity of factors such as lack of availability of public health services, higher charges by private health care providers and the household’s low ability to pay and deprivation in the non-economic domain. Besides income, education acts as an efficiency parameter in improving health. Those who are multidimensionally poor are more likely to be deprived both in material and non-material dimensions leading to poor health and high catastrophic health spending.

Fifth, health shocks such as the death of family members and frequency of sickness of family members along with multidimensional poverty are significant predictors of catastrophic health spending. While demographic characteristics of households are not consistently significant across the country of residence, health shocks are significant irrespective of the country of residence.

The high health spending among the multidimensionally poor may be due to a number of factors including higher prevalence of morbidity among the multidimensionally poor, lack of accessibility and availability of public health services, and high private health care costs. Thus, the multidimensionally poor may resort to selling assets and borrowing to meet health care needs, which may aggravate the poverty level of the household. In this context

- The social determinants of health that recommend health status as a concern to all policy makers and not merely within health sectors assume significance [51]. Policies that aimed to improve health need to focus on reduction of poverty, improving nutrition and education of the population.
- Improvements in the circumstances in which people are born, work, live and work are necessary to reduce the ill health of the poor and high health spending [52].
- Public spending on health (central, state and local governments) attains greater significance. Increasing government investment in health infrastructure and increasing budgetary allocation on health can reduce out-of-pocket expenditure and catastrophic health spending in the population.
- Increasing access to health insurance to households will be useful to reduce the extent of catastrophic health spending.

Besides, increasing investment in education, employment generation and improved water and sanitation in each of these regions will certainly reduce the extent of multidimensional poverty and may reduce catastrophic health spending. We also suggest further research on understanding the type of health morbidity and cause of higher health spending in these poorer regions.

We acknowledge some limitations owing to data constraints. The PVA survey has limited information on the type of morbidity and health; type of medical expenditure and is based on the respondent’s own assessment of the situation. Hence, we could not link health care utilization and the type of morbidity among the multidimensionally poor and the multidimensionally non-poor and details of expenditure on medicine, tests, hospital charges etc. These surveys have a specific mandate for certain geographical regions and so they are not national estimates. However, the surveys have the advantage that they were not aimed at assessing the health related aspects, therefore, the answers provided by respondents and recorded by the surveyor have the least chance of any intentional and professional bias. The health expenditure data were collected in a reference period of one year and may have been underestimated to a limited extent.

## Conclusion

The findings underscore that the multidimensionally poor are more likely to face health shocks and incur catastrophic health spending irrespective of the country of residence. Not only do the multidimensionally poor incur high catastrophic health spending, they also face long-term impoverishment and untreated morbidity. Global, regional and national policies should be oriented/sensitive to reduction of multidimensional poverty and the extent of catastrophic expenditure. Reduction of multidimensional poverty will certainly help to reduce the extent of catastrophic health spending.

## Abbreviations

AF: Alkire and foster
CTP: Capacity to pay
ICIMOD: International Center for Integrated Mountain Development
HICAP: Himalayan Climate Change Adaptation Programme
HKH: Hindu Kush Himalayan
MDG: Millennium Development Goals
MPCE: Monthly per-capita consumption expenditure
MPI: Multidimensional poverty index
NCD: Non-communicable diseases
OOPE: Out-of pocket expenditure
Pl: Poverty line
PVA: Poverty and vulnerability survey
SDG: Sustainable development goals
SES: Socioeconomic status
UN: United Nations
UNDP: United Nation Development Program
WHO: World Health Organization

## Footnotes

1 The HKH region include eight countries - Afghanistan, Bangladesh, Bhutan, China, India, Myanmar, Nepal and Pakistan

2 As of 2011, India consists of 35 states/union territories, Nepal consists of 6 regions and Myanmar consists of 14 state/regions
